# A postoperative left ventricular-right atrial shunt due to infectious endocarditis after aortic repair with aortic valve replacement detected by transesophageal echocardiography

**DOI:** 10.1186/s40981-016-0052-7

**Published:** 2016-09-29

**Authors:** Toshiyuki Sawai, Junko Nakahira, Manabu Kitano, Toshiaki Minami

**Affiliations:** Department of Anesthesiology, Osaka Medical College, 2-7 Daigaku-machi, Takatsuki, 569-8686 Japan

## Abstract

Infectious endocarditis (IE) with acute heart failure is a medical emergency. In particular, postoperative IE after aortic repair with an artificial vascular graft is a life-threatening matter. We present a case in which a mobile abscess appeared on the aortic valve annulus with an intra-cardiac shunt in the left ventricle (LV) to the right atrium (RA) after ascending aortic repair with aortic valve replacement (AVR) for acute type A aortic dissection. It was diagnosed with transesophageal echocardiography (TEE), which prompted further exploration.

## Background

Intra-cardiac shunts in the left ventricle (LV) to the right atrium (RA) are mostly congenital [1]. Similar acquired shunts are reported as follows: chest trauma, mitral and aortic valve replacement, or infectious endocarditis (IE) [2, 3]. In particular, intra-cardiac shunts with periannular abscesses related to IE have been reported in 14 % of patients after prosthetic valve replacement [2]. We present a case in which a mobile abscess appeared on the aortic valve annulus with an intra-cardiac shunt in the LV to the RA after ascending aortic repair with aortic valve replacement (AVR) for acute type A aortic dissection. It was diagnosed with transesophageal echocardiography (TEE), which prompted further exploration.

## Case presentation

**Fig. 1:**
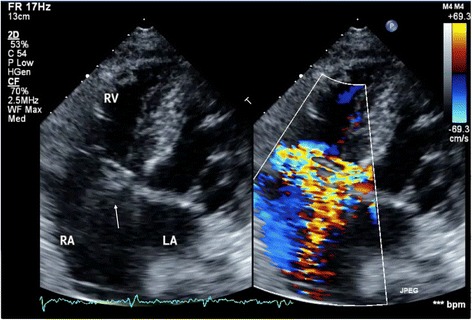
Images of transthoracic echocardiography (TTE) in the apical four-chamber view. *Left* two-dimensional TTE, demonstrating the vegetation attached to the annulus of the aortic valve (*arrow*). *Right* color Doppler TTE, demonstrating the abnormal flow into the right atrium

**Fig. 2:**
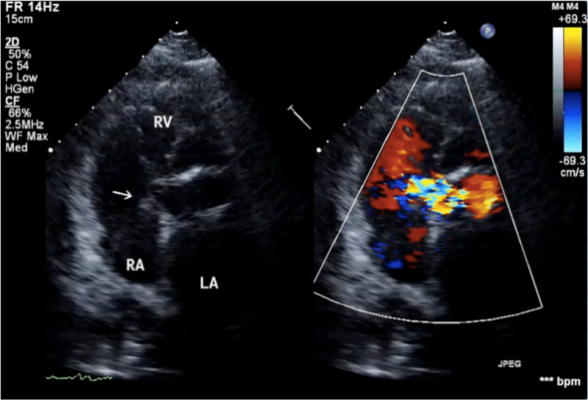
Transthoracic echocardiography (TTE) at the parasternal aortic short axis view. *Left* two-dimensional TTE, demonstrating the vegetation attached to the annulus of the aortic valve (*arrow*). *Right* color Doppler TTE, demonstrating the abnormal flow into the right atrium

**Fig. 3:**
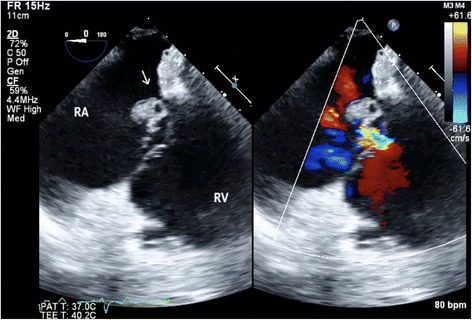
Transesophageal echocardiography (TEE) in the mid-esophageal modified four-chamber view. *Left* two-dimensional TEE, demonstrating the vegetation attached to the annulus of the aortic valve (*arrow*). *Right* color Doppler TEE, demonstrating the abnormal flow into the right atrium

**Fig. 4:**
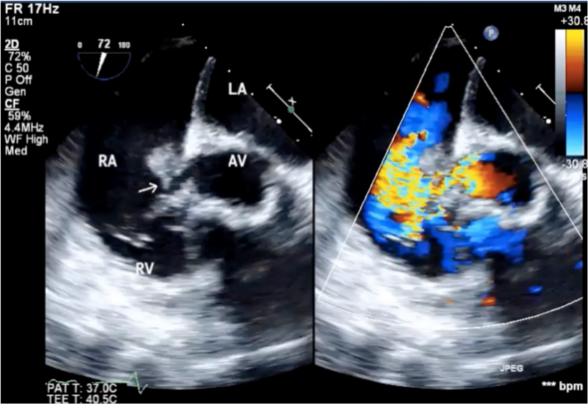
Images of transesophageal echocardiography (TEE) in the mid-esophageal modified aortic valve short axis view. *Left* two-dimensional TEE, demonstrating the vegetation attached to the annulus of the aortic valve (*arrow*). *Right* color Doppler TEE, demonstrating the abnormal flow into the right atrium through the subaortic valve

**Fig. 5:**
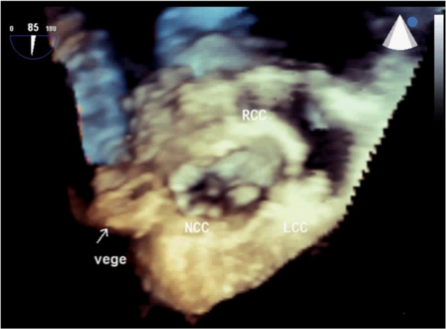
Three-dimensional imaging structured with transesophageal echocardiography. This revealed the exact location of the vegetation attached to the aortic root structures

An 82-year-old woman underwent emergent aortic repair for acute type A aortic dissection. Intraoperative TEE showed aortic dissection in ascending to abdominal aorta with moderate aortic valve (AV) regurgitation. Neither regional wall motion abnormalities nor tricuspid and mitral valve abnormalities were shown on TEE. Under cardiopulmonary bypass (CPB), the aortic valve was replaced with a biological valve. After that, under circulatory arrest with selective brain perfusion, the ascending aorta was replaced with a vascular graft. The TEE after weaning from CPB showed no unusual findings. Tracheal extubation was performed on postoperative day (POD) 1. The postoperative course was comparatively stable and inflammatory reaction was not remarkable. We administrated an antibacterial drug, cefazolin of 2 g intraoperatively and 1 g/day postoperatively. On POD 9, however, she suddenly complained of both dyspnea and fever. C-reactive protein (CRP) was remarkably increased at 22 mg/dl. To prevent infection of the artificial vascular graft, we changed the antibacterial drug, vancomycin of 1.5 g/day and imipenem of 0.5 g/day. She was transferred to the intensive care unit (ICU) immediately. After emergency tracheal intubation, we inserted the pulmonary artery catheter (PAC) from the right internal jugular vein to evaluate cardiac function. Mixed venous blood oxygen saturation obtained from the PAC was revealed to be 90 %. As we suspected an intra-cardiac shunt, we firstly performed a transthoracic echocardiography (TTE). The two-dimensional TTE in the apical four-chamber view showed that a large vegetation seemed to be attached on the septal leaflet of the tricuspid valve (Fig. 1). Furthermore, TTE with color Doppler from a parasternal aortic valve short axis view showed abnormal flow in the RA (Fig. 2). To evaluate intra-cardiac appearance in detail, we performed a TEE examination, suspicious of an intra-cardiac shunt. The two-dimensional TEE from the modified four-chamber view clearly showed a large vegetation on the annulus of the aortic valve which protruded into the RA chamber (Fig. 3). Furthermore, the color Doppler TEE from the modified five-chamber view showed an intra-cardiac shunt from the LV to the RA through a subaortic ventricular septal fistula (Fig. 4). The three-dimensional TEE showed a structural connection between the aortic valve annulus and the attachment site of the vegetation (Fig. 5). After the intraoperative TEE found the abnormalities coincidently, we decided to perform an emergency surgical repair. We performed aortic annuloplasty with re-AVR and closure of the subaortic ventricular septal fistula using bio-pericardium. After repairing, the patient was somehow weaned from CPB with a high-dose inotrope. We confirmed by TEE that there was no pathologic flow associated with the fistula. Pathogenic bacteria were gram-positive coccus. In spite of all our medical efforts, on 30 POD, the patient died of multiple organ failure due to IE at the hospital.

### Discussion

**Fig. 6:**
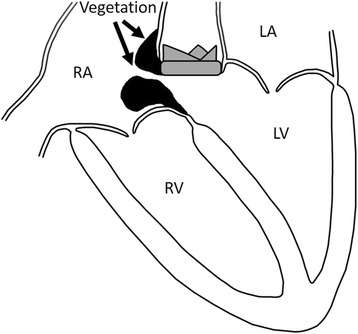
A schematic drawing of the present case. The vegetation was attached to the aortic valve annulus and the subaortic structure. There is an intra-cardiac shunt from the left ventricle to the right atrium through a subaortic ventricular septal fistula

An acquired ventricle-atrium shunt has various etiologies: (1) after aortic and/or mitral valve replacement surgery, (2) infective endocarditis, and (3) chest trauma [4]. A schematic transverse section of a heart ﻿of the case is shown in Fig. 6; a membranous ventricular septum divides the LV chamber from the RA chamber [5]. Because a membranous ventricular septum is structured by thin connective tissue of the fibrous ring, an acquired LV-RA and/or RV shunt is likely to generate a membranous ventricular septum. The authors thought that the membranous ventricular septum was included in the aortic prosthetic valve structure line because of its proximity to the aortic root, resulting in IE and subsequently resulting in intra-cardiac shunt.

After the patient complained of the abnormality, the authors firstly inserted the PAC to evaluate the cardiac function. This revealed abnormal increased mixed venous blood oxygen saturation. Among many causes of increased mixed venous blood oxygen saturation, an intra-cardiac left to right shunt is one of the possible causes because of acute appearance of dyspnea. Although the patient unfortunately died of acute heart failure due to IE, decisive diagnosis without delay was required for performing adequate treatment. Although TTE revealed both the vegetation on the peri-aortic root and abnormal flow in the RA, the detailed site of the intra-cardiac shunt was difficult to detect. Because TEE depicts the subaortic structures clearly using an acoustic window from both of the LA and the RA, TEE depicts the structures better than TTE [6]. One of the tips for clearly depicting the detailed site of the LV-RA shunt is to adjust the aliasing velocity appropriately [7]. The authors adjusted the aliasing velocity at 30.8 cm/ms. Because pressure gradient between the LV and the RA decreases due to pressure overload at the RA, a relative slow velocity of shunt flow was predicted. Furthermore, three-dimensional TEE revealed the exact location of the vegetation attached to the aortic root structures. In this aspect, TEE examination in the ICU was a crucial diagnostic method to decide emergent surgical revision.

## Conclusions

Although the patient died of acute heart failure due to infectious endocarditis, TEE information could be helpful for depicting the exact location of the fistula and for guiding the surgical procedure.

## Abbreviations

AV: Aortic valve
LA: Left atrium
LCC: Left coronary cusp of the aortic valve
LV: Left ventricle
NCC: Noncoronary cusp of the aortic valve
RA: Right atrium
RCC: Right coronary cusp of the aortic valve
RV: Right ventricle
TV: Tricuspid valve
vege: Vegetation
